# Boiling and quenching heat transfer advancement by nanoscale surface modification

**DOI:** 10.1038/s41598-017-06050-0

**Published:** 2017-07-21

**Authors:** Hong Hu, Cheng Xu, Yang Zhao, Kirk J. Ziegler, J. N. Chung

**Affiliations:** 1 Cryogenics Heat Transfer Laboratory, Department of Mechanical and Aerospace Engineering, University of Florida, Gainesville, FL 32611-6300 USA; 2 Nanostructured Interfaces Laboratory, Department of Chemical Engineering, University of Florida, Gainesville, FL 32611-6005 USA

## Abstract

All power production, refrigeration, and advanced electronic systems depend on efficient heat transfer mechanisms for achieving high power density and best system efficiency. Breakthrough advancement in boiling and quenching phase-change heat transfer processes by nanoscale surface texturing can lead to higher energy transfer efficiencies, substantial energy savings, and global reduction in greenhouse gas emissions. This paper reports breakthrough advancements on both fronts of boiling and quenching. The critical heat flux (CHF) in boiling and the Leidenfrost point temperature (LPT) in quenching are the bottlenecks to the heat transfer advancements. As compared to a conventional aluminum surface, the current research reports a substantial enhancement of the CHF by 112% and an increase of the LPT by 40 K using an aluminum surface with anodized aluminum oxide (AAO) nanoporous texture finish. These heat transfer enhancements imply that the power density would increase by more than 100% and the quenching efficiency would be raised by 33%. A theory that links the nucleation potential of the surface to heat transfer rates has been developed and it successfully explains the current finding by revealing that the heat transfer modification and enhancement are mainly attributed to the superhydrophilic surface property and excessive nanoscale nucleation sites created by the nanoporous surface.

## Introduction

## Importance of boiling and quenching heat transfer

All power production, refrigeration, and advanced electronic systems depend on efficient cooling mechanisms for the purpose of achieving higher power density and higher system efficiency. Among the three heat transfer modes, convective heat transfer, especially coupling with a liquid-vapor phase change process such as boiling and condensation, has been traditionally relied on to provide the super high cooling rates for high power density systems such as nuclear reactors and high performance electronic devices. However, as engineers continue to push the advanced power devices such as supercomputers, Generation IV nuclear reactor concepts and NASA’s new heavy lifting rockets for more powerful functions and also to conserve energy consumption, correspondingly, higher and higher system operating temperatures and power densities are becoming the goals of future energy system design. Accordingly, researchers are turning to untraditional and revolutionary convective thermal energy transport mechanisms for solutions as the conventional boiling heat transfer technologies have reached their limits. In this research, we report the potential and feasibility of nanoscale textured heat transfer surface to provide such a possible solution.

In many convective liquid-vapor phase change heat transfer applications, cryogenic fluids are widely used in industrial processes, space exploration, and cryosurgery systems and so on. For example, cryogens are usually used as liquid fuels such as liquid hydrogen and oxygen in the rocket industry, liquid nitrogen and helium are frequently used to cool superconducting magnetic device for medical applications. In these systems, proper transport, handling, and storage of cryogenic fluids are of great importance. When a cryogenic system is first started up, its walls and hardware must go through a transient chilldown period prior to reaching a steady operation. Therefore, chilldown (quenching) is the process of keeping the system adjusted to the low temperature scale which is usually several hundred degrees below the room temperature. The chilldown or quenching process is complicated, involving unsteady two-phase heat and mass transfer, and has not been fully understood.

## Characteristics of boiling and quenching heat transfer

**Figure 1:**
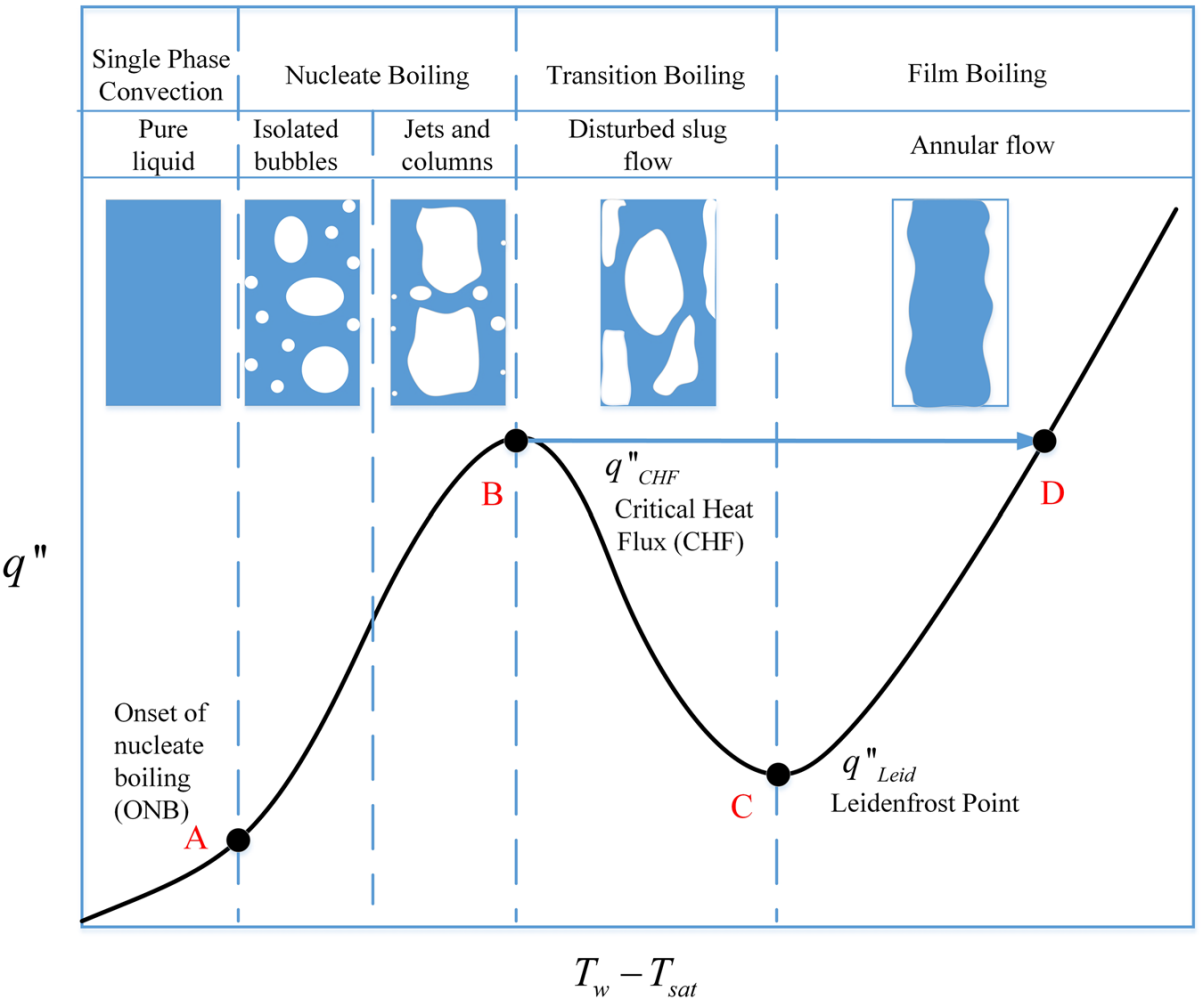
A typical boiling curve.

The liquid-vapor phase change heat transfer process is characterized by a “curve” that shows the functional relationship between the heat transfer surface heat flux and the surface temperature. Figure 1 is a typical such curve where the heater surface heat flux, *q*″, is plotted against the heater surface degree of superheating, *T*_*W*_ − *T*_*sat*_, where *T*_*W*_ is the surface temperature and *T*_*sat*_ is the saturation temperature corresponding to the flow system pressure. In boiling, the heating is externally supplied to the heater surface and thus, heat input can be controlled independently, such as the constant wall heat flux condition. In this case, boiling is a heat flux (independent variable) controlled process. For the heat flux controlled condition, as the heating rate is progressively increased, the process follows the route of A → B → D. In order to avoid a huge temperature jump from B → D, boiling applications usually run safely below point B in the nucleate boiling regime for nuclear power plants.

In contrast, during quenching the heat transfer wall does not have an external source of heat supply, therefore, the heat transferring out of the wall can only be supplied internally from the thermal capacity (stored energy) of the wall. The only way to remove heat from the wall is by lowering the inner wall surface temperature using a cooling flow. Accordingly, the wall surface temperature is the independent variable and also the control parameter in quenching. In summary, quenching, a wall temperature-controlled process, is a conjugate process where the rate of heat transfer is controlled by the surface temperature variation. The rate of heat transfer to the fluid can only be associated with the temperature change of the wall inner surface. As a result, the wall surface temperature is the controlling parameter that forces the quenching process to follow the route D → C → B → A.

In boiling applications, such as that in the cooling of a nuclear reactor, taking advantage of a super high flux of heat transfer in the nucleate boiling regime with a relatively low wall surface temperature is a standard practice. The only precaution is not to exceed the critical heat flux (CHF) as any boiling process that goes beyond the CHF would enter the film boiling directly that results in a huge wall temperature jump and the burnout (melting) of the heater wall (nuclear reactor fuel rod). In the past decade, many research efforts have focused on raising the CHF limit for safer and higher heat flux boiling applications. However, film boiling is the first mode of heat transfer encountered in most quenching processes, and it usually cannot be avoided. Due to its low heat fluxes at high wall temperatures, the quenching efficiency, in general, is extremely low. According to Shaeffer *et al*.^1^, the average quenching efficiency that is defined as the ratio of the amount of thermal energy removed from the wall versus the maximum cooling capability of the cryogen spent in a phase change process is about 8%, highlighting the tremendous need to improve the quenching efficiency for many applications that require a cryogen as the working fluid. The key concept to increase the efficiency is to shorten the film boiling by raising the Leindenfrost temperature (point C in Fig. 1).

## Contact angle and wettability modifications by nanoscale surface textures

The majority, if not all, of the boiling heat transfer enhancement attempts were focused on the nucleate boiling regime and the CHF. There have been a number of experiments aimed at measuring the contact angle and wettability on nanoporous surfaces. Luo’s group^2–4^ have introduced more detailed discussion both theoretically and experimentally on the wettability of surfaces with nano pillars and derived an angle criterion for determining transition from Cassie-Baxter to Wenzel states. Singh *et al*.^5^ investigated the wetting and evaporation of sessile droplets on nanoporous anodic aluminum oxide (AAO) substrates having different pore distribution (uniform, random and linearly arranged) morphologies and pore sizes (70–120 nm). They claimed that a nanoscale structured surface is an applicable tool to control wettability as well as the diffusive evaporation process. In addition, physical morphology and pore distribution affect wettability as well as evaporation rates. Tasaltin *et al*.^6^ made nanoporous AAO surfaces having a hexagonal structure of circular pores with 250 nm in diameter and 300 nm in depth and found that the surfaces to be superhydrophobic. The contact angle was measured at 153.2 ± 2° for vapor-phase hexamethyldisilazane (HMDS), that is larger than the average water contact angle of 82.9 ± 3° on smooth thin film AAO surfaces. The excellent surface wettability associated with these nanoporous surfaces has a significant potential to enhance boiling heat transfer.

Liter and Kaviany^7^ reported a nearly three times improvement to heat transfer for a porous-layer coating over a conventional surface. Arik *et al*.^8^ reported an experimental study finding that microporous, diamond-coated surfaces provided an average 1.6 enhancement factor on the critical heat flux (CHF) of FC-72 pool boiling.

Li *et al*.^9^ reported an experimental study that investigated the CHF of a high-velocity circular water jet impingement boiling on the stagnation zone of a nano-structured heater surface with different surface wettability parameters. The wettability of the copper heater was modified with nanostructures to change the surface topography and chemistry to exhibit either a hydrophilic or hydrophobic character. They found that the wettability holds a strong influence on the CHF. For example, the CHF almost doubled when the contact angle was reduced from 105° to 5°. Hsu and Chen^10^ experimentally investigated the effects of surface wettability on water pool boiling heat transfer. They used coatings of nano-silica particles to change the wettability of the copper surface from superhydrophobic to superhydrophilic. They demonstrated that CHF values increased from 300 to 1483 kW/m^2^ when the contact angle decreased from 145° (hydrophobic) to less than 10° (hydrophilic).

O’Hanley *et al*.^11^ experimentally evaluated the separate effects of surface wettability, porosity, and roughness on the CHF of water using engineered surfaces. They reported that porous hydrophilic surfaces enhanced CHF by 50–60%, while porous hydrophobic surfaces resulted in a reduction of CHF by 97%. Phan *et al*.^12^ also focused on the influence of surface wettability on nucleate boiling heat transfer. They used nanocoating techniques to vary the water contact angle from 20° to 110°. They reported that a greater surface wettability due to the hydrophilic nature of the surface increases the vapor bubble departure radius and reduces the bubble departure frequency. The best heat transfer coefficients were obtained for the surfaces that had a water contact angle close to either 0° or 90°.

Hens *et al*.^13^ used molecular dynamics simulations to study the boiling mechanisms of argon bubble formation on a platinum substrate with a particular emphasis on the effects of nanosized textures on the surface. The bubble nucleation and vapor film formation showed a dependence on the degree of superheat and solid–liquid interfacial wettability since bubbles did not form easily on non-wetting surfaces. They concluded that a wettable (hydrophilic) surface facilitates higher heat transfer.

The only study found in the literature that addressed the effects of nano-textured surfaces on the Leidenfrost temperature is given by Kruse *et al*.^14^. The micro- and nano-structures were fabricated on a stainless steel surface by a femtosecond laser. The traditional Leidenfrost droplet experiment was performed on both machine-polished and nano-textured surfaces. They observed an extraordinary shift in the Leidenfrost temperature for water from 280 °C for the machine-polished surface to 450 °C for the nano-textured superhydrophilic surface. The gigantic 175 °C increase in the Leidenfrost temperature was attributed to a reduction in the contact angle that results in intermittent liquid/solid contact and capillary wicking action.

## Wall heat flux controlled boiling heat transfer

Nanowires have been found to alter the heater surface heat transfer properties that enhance boiling heat transfer performance. Using Si and Cu nanowire arrays coated surfaces, Chen *et al*.^15^ and Lu and Kandlikar^16^ discovered enhancement in the nucleate boiling regime, and in particular, more than 100% increases over the conventional plain surfaces in the critical heat fluxes were found in the pool boiling experiment with deionized water. Also using nanowire coated surfaces, Li *et al*.^17^ measured flow boiling performance in microchannels for electronics cooling applications. They reported 15–20% increases in the nucleate boiling heat transfer rates. Ahn *et al*.^18^ and Sathyamurthi *et al*.^19^ reported experimental results of pool boiling in PF5060 fluid on silicon heaters coated with vertically aligned, multi-walled carbon nanotubes (MWCNTs). Type A and Type B NWCNTs have layer thicknesses of 9 μm and 25 μm, respectively. They found that the critical heat flux was increased by 62% and 58% using Type A and Type B, respectively. With the Type B heater, the minimum heat flux at the Leidenfrost point was increased by 150%. It is believed that the nano-sized film evaporation contributes to the super high heat flux transport rates. Lu and Kandlikar^16^ have provided a comprehensive review on the nanoscale surface modification techniques for pool boiling enhancement. The authors emphasized that since the available literature on boiling from a nano-structured surface is exclusively experimental in nature and contains controversies, as a result the underlying physical mechanisms that cause the heat transfer enhancements are not fully understood. However, the review suggested that the nanoscale surface modifications could be responsible for the changes in surface properties such as wettability and contact angles that results in the boiling improvements. However, the authors concluded that the actual physical mechanisms are not completely clear.

Vemuri and Kim^20^ experimentally investigated the pool boiling heat transfer from nano-porous surface immersed in a saturated FC-72 dielectric fluid at atmospheric pressure (101 kPa). The data obtained from nano-porous surface (Anodisc 25) of thickness about 70 µm and pores in the range of 50 nm to 250 nm made from aluminum oxide (Al2O3) were compared to that of a plain surface (aluminum) of thickness about 105 Am. The authors found that there is a reduction of about 30% in the incipient superheat and an 80% increase in heat flux for the applied power levels for nano-porous surface over a plain surface.

Lee *et al*.^21^ have used the anodizing technique to grow the well-ordered oxide nanostructures on a metal substrate. In their experimental study, the nucleate pool boiling heat transfer coefficient and boiling incipient wall temperature were investigated with water as the working fluid. The incipient wall superheats of pool boiling on the nano-porous surface was lower than that on the non-coating surface by 2–3 K. The nucleate boiling heat transfer coefficients of nano-porous coating surface were higher than those of non-coating surface by about 10% when the heat flux is lower than 60 kW/m^2^. The improved pool boiling heat transfer was attributed solely to the nanoscale porous surface layer.

## Wall temperature controlled quenching heat transfer

Compared to the wall heat flux controlled boiling case, the amount of literature on quenching heat transfer is far less. For convective quenching in tubes and pipes, most of them were using water and normal temperature fluids^22–24^. Yuan and Chung^25^ reported liquid nitrogen chilldown in conventional stainless tubes under low flow rate. There is no convective quenching work with surface modification found in the open literature. For the quenching in a water pool environment, Vakaraelski *et al*.^26^ investigated heat transfer for four different surface modifications that result in hydrophobic, superhydrophobic, hydrophilic and superhydrophilic. However, cryogenic fluids are quite different from water. Hu *et al*.^27^ is the first to perform liquid nitrogen quenching experiment using a nano-structured surface and proved its feasibility for enhancing heat transfer over the normal surface. The current work not only extended the liquid nitrogen quenching experiment by Hu *et al*.^27^ with much-improved nanoscale surface textures but also expanded the scope to cover heated surface boiling experiment with surface nanoscale textures.

## Result

### Characterization of nanoporous test heater surface

**Figure 2:**
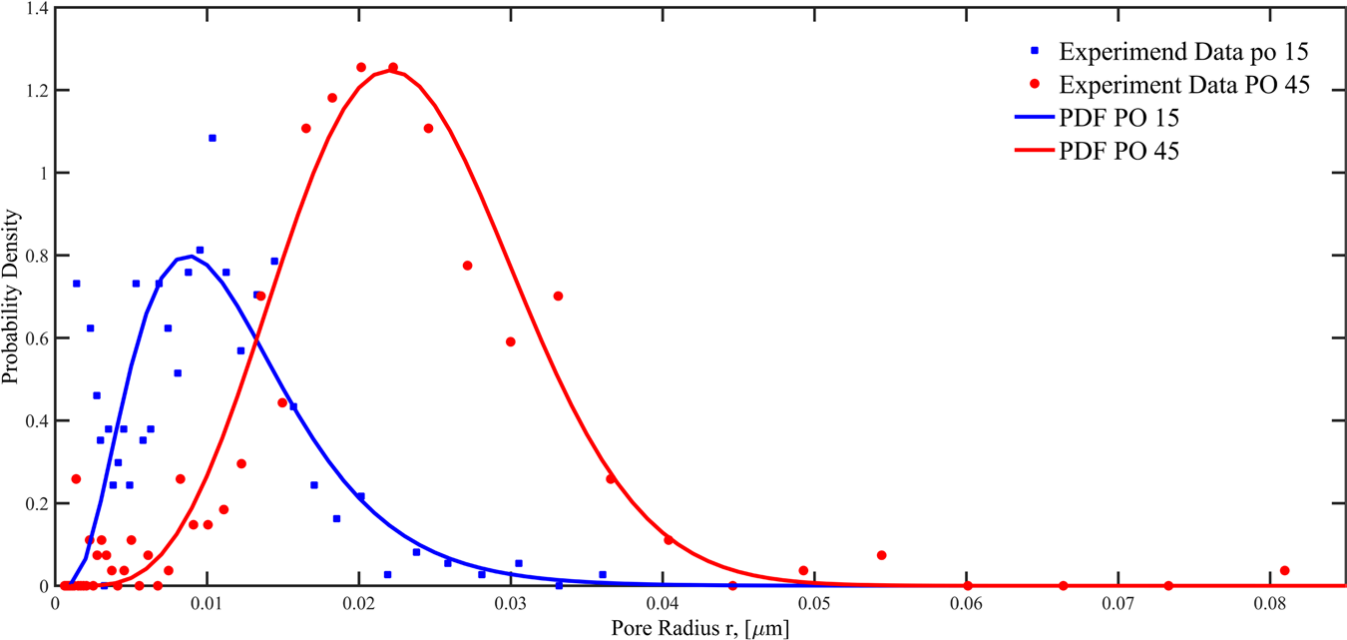
Nanoporous surface cavity probability density distributions for both PO15 and PO45 surfaces.

Based on the experimental data, Weibull function was used to fit the distribution of the nanopore radius. Table 1 lists the parameters of probability density function on the nanopore radius on the nanoporous surface and Fig. 2 shows the probability density functions on nanopore radius for both Po15 and PO45 surfaces, respectively.

**Table 1:** Nanoporous surface pores characteristics.

	PO 15	PO 45
λ, the scale parameter	2.529	3.211
k, the shape parameter	5.272	10.84
Mean pore radius (µm)	0.008	0.019
Number density (1/cm^2^)	2.0E10	1.24E10

As Fig. 2 shows, the PO45 surface has nanopores with a wider range in size than those of the PO15 surface. The respective mean pore radius are 19 nm and 8 nm for PO45 and PO15, respectively.

**Figure 3:**
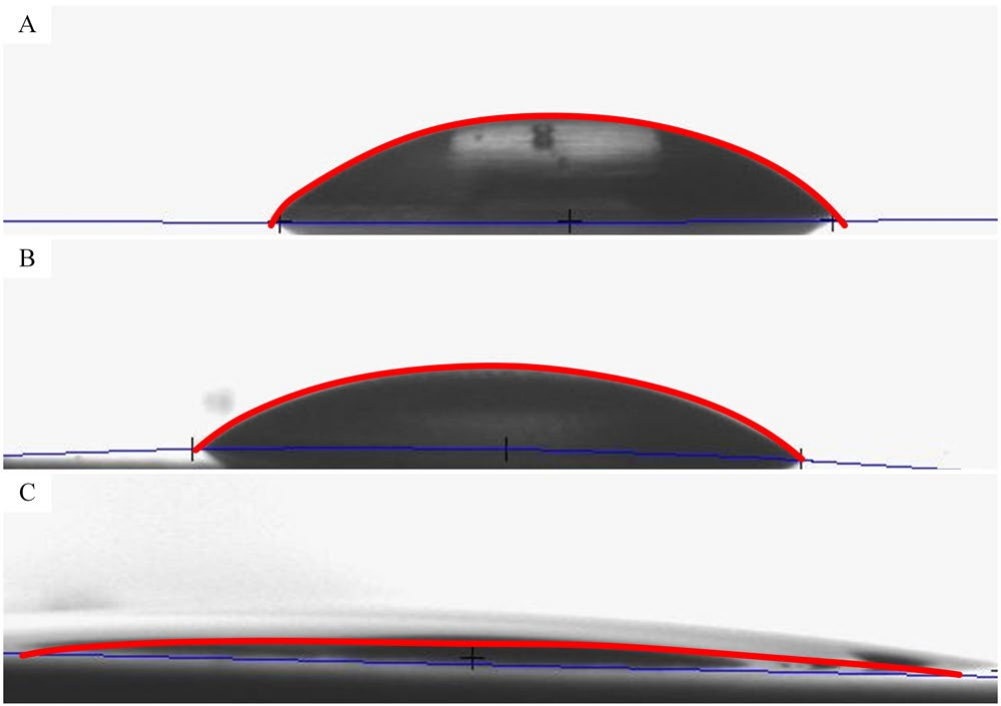
Contact angles on (**A**) Normal machine polished, (**B**) PO 15 and (**C**) PO 45 surfaces are measured to be 46.2 ± 1.5°, 38.6 ± 1.2° and 7.4 ± 0.2°, respectively.

Furthermore, the contact angles for water in air have been measured on both PO15 and PO45 surfaces in addition to nanopore size analysis. Figure 3 shows the differences in contract angles for the 6061 multipurpose aluminum alloy surfaces. The average contact angle on the normal, PO15 and PO45 surface are 46.2 ± 1.5°, 38.6 ± 1.2° and, 7.40 ± 0.2° respectively.

Li *et al*.^28^ used numerical modeling verified by experimental data to suggest that liquid nitrogen contact angle is less than 7.5° on normal metal surfaces. As a result, we have assumed that the liquid nitrogen contact angle on the normal surface is 7.5°. Then we used the same proportional reduction ratios of water on P15 and PO45, respectively to estimate the liquid nitrogen contact angles on PO15 and PO45. The estimated contact angles for liquid nitrogen are listed in Table 2. These estimated liquid nitrogen contact angles were used in the analysis to calculate the active nucleation site radii and total contact line lengths in the Methods section on the subject of “Modeling of Maximum and Minimum Active Nucleation Cavities”.

**Table 2:** Measured contact angles for water and estimated contact angles for LN2.

	Normal	PO15	PO45
Contact angle measured in water (°)	46.2	38.6	7.40
Uncertainty of contact angle measured in water (°)	±1.5	±1.2	±0.2
Estimated contact angle for liquid nitrogen (°)	7.50	6.27	1.20

### Quenching heat transfer characteristics

**Figure 4:**
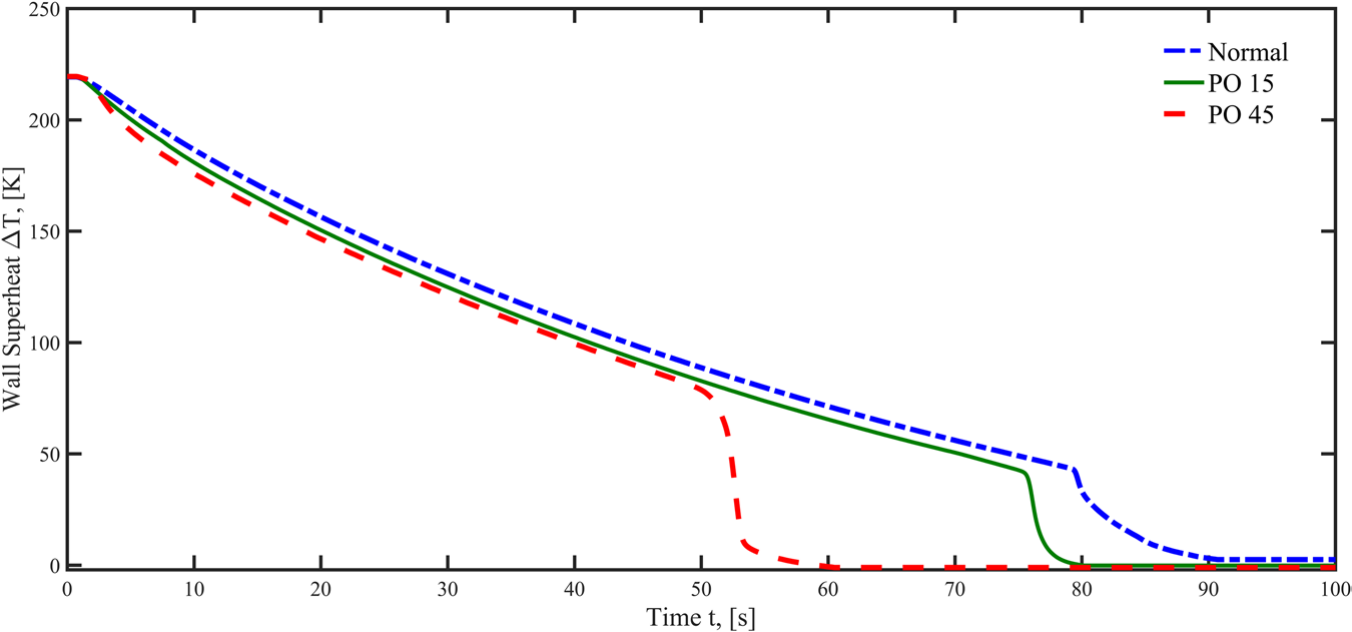
Quenching curves for the three experimental surfaces.

The quenching (chilldown) curve, that shows the test surface temperature history during the transient process, is plotted in Fig. 4. The PO45 surface produced the shortest quenching time at 61 seconds, however, PO15 and the normal surfaces required 80 seconds and 91 seconds, respectively. In Fig. 4, the chilldown time is also shorter for the PO45 nanoporous surface (61 s) than for the normal surface (91 s) by approximately 33%. If we assume the same mass flow rates are used to chill the two pipes with identical dimensions, wall thickness, and material, it is reasonable to assume that the pipe with a nanoporous inner surface would save approximately 33% in cryogenic fluid consumption.

The reducation of the chilldown time is chiefly attributed to the shorter film boiling regime. The main reason for this is that the PO45 surface possesses the most organized and established a nanoporous structure that results in more active nucleation sites. More detailed analysis and discussion on the effects of nanoporous structure on the nucleation sites and the corresponding nucleation rates are given later. Furthermore, all three surfaces have the same quenching (heat transfer) rate in the film boiling regime. This phenomenon confirms the film boiling transport mechanism is not affected by the surface condition.

**Figure 5:**
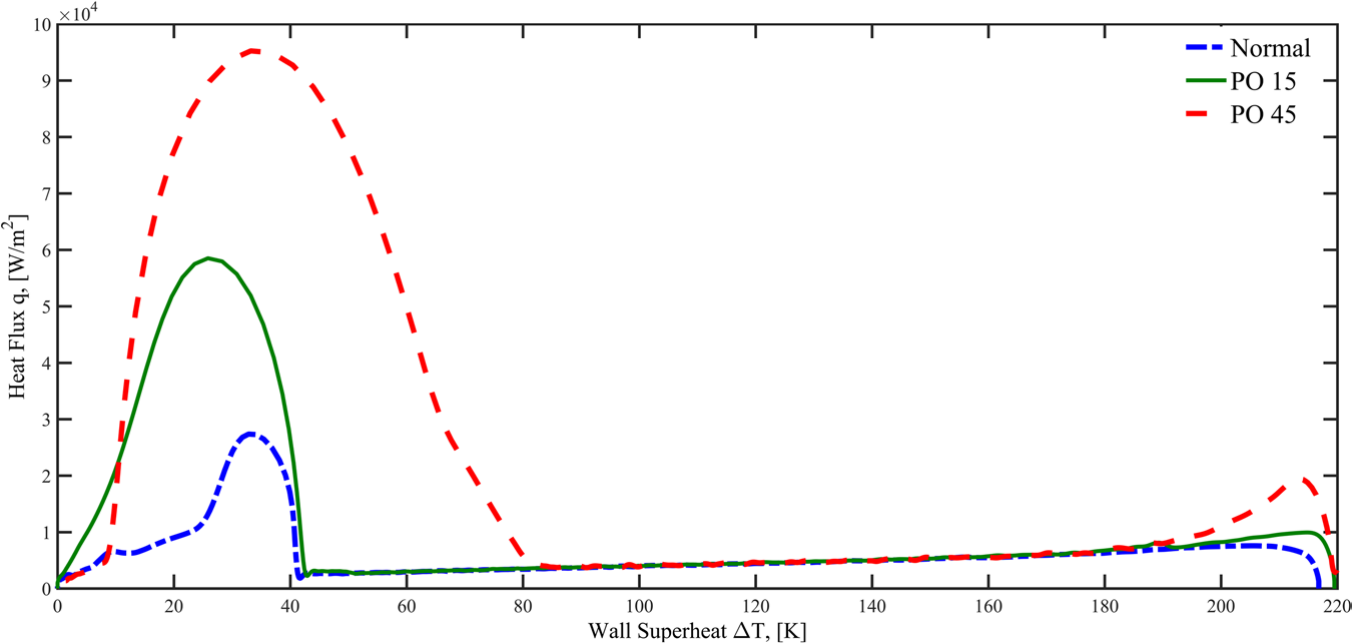
Boiling curves for the three surfaces during quenching experiments.

The so-called boiling curve (heat flux vs. degree of surface superheat) during chilldown experiment is plotted in Fig. 5. In Fig. 5, the three curves overlap in the film boiling regime until they each turn into transition boiling at different Leidenfrost temperatures. The Leidenfrost point for the PO 45 surface is at 160 K while those for the PO15 surface and normal surface are at 121 K and 120 K, respectively. In another word, the PO45 surface was able to raise the Leidenfrost temperature about 40 K over the other two surfaces that basically facilitated the shortest quenching time of 61 seconds.

### Boiling heat transfer characteristics

**Figure 6:**
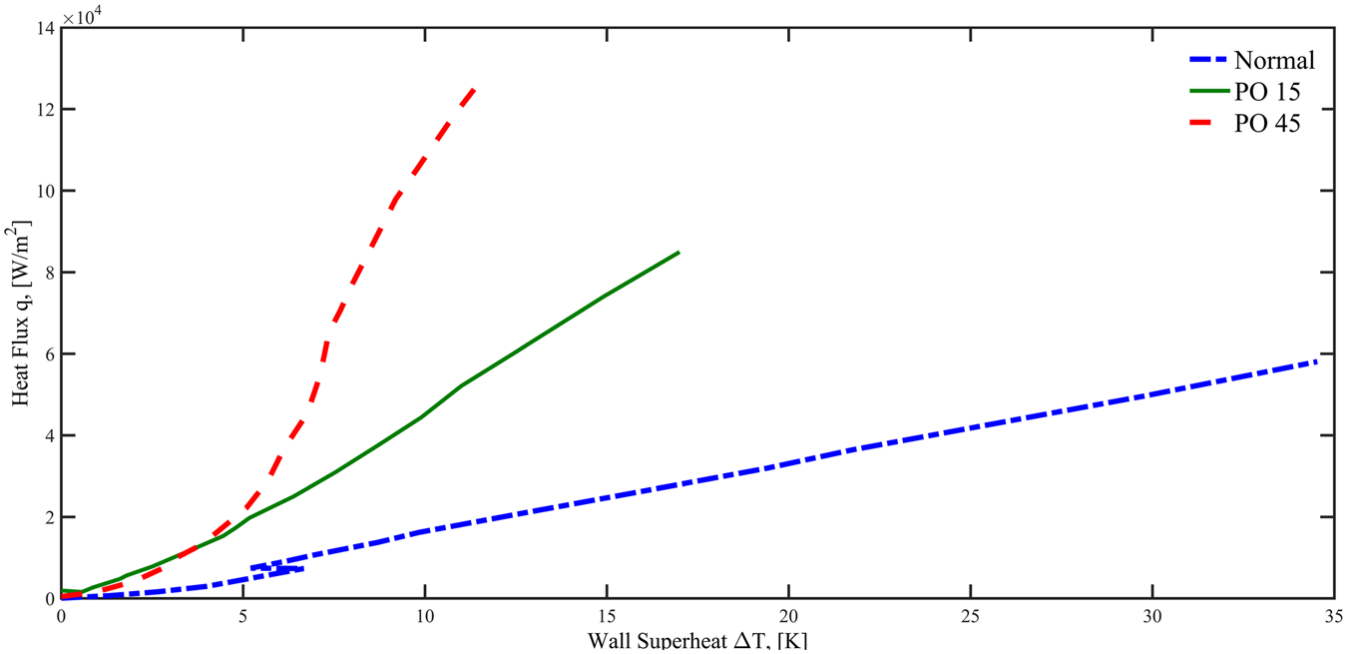
Boiling curves for the three surfaces during heated boiling experiments.

In addition to the quenching experiment, the forward boiling experiment where the test section was heated during the experiment, was also performed. In Fig. 6, three curves show the same general boiling trends that the heat fluxes increase with increasing heater (test surface) superheat (surface temperature minus the saturation temperature). Compared with the normal surface, the PO45 surface has the highest rate of heat flux increase followed by the PO15 surface. The end point of the curve represents the critical heat flux (CHF). The PO45 surface possesses the highest CHF value at about 1.25 × 10^5^ W/m^2^. The PO 15 surface is the next with 8.3 × 10^4^ W/m^2^ and the normal surface is the lowest with 5.9 × 10^4^ W/m^2^. With respect to the wall superheats on heater surfaces at the CHF point, the PO45 surface has the smallest with 12 K while the superheats for PO15 and the normal surfaces are 18 K and 35 K, respectively.

The nanoporous cavities are able to enhance the boiling efficiency by raising the CHF value while reducing the heater surface superheat. In other words, the nanoporous surface can provide much higher heat transfer coefficients in the nucleate boiling regime. For example, the heat transfer coefficients for the traditional, PO15, and PO45 surfaces at the CHF point are, 1675 W/m^2^K, 4732 W/m^2^K and 10640 W/m^2^K respectively. Also, the average heat transfer coefficients in nucleate boiling for the traditional surface, PO15, and PO45 were measured at 1296 W/m^2^K, 2617 W/m^2^K, and 6599 W/m^2^K, respectively.

### Boiling heat transfer visualization

**Figure 7:**
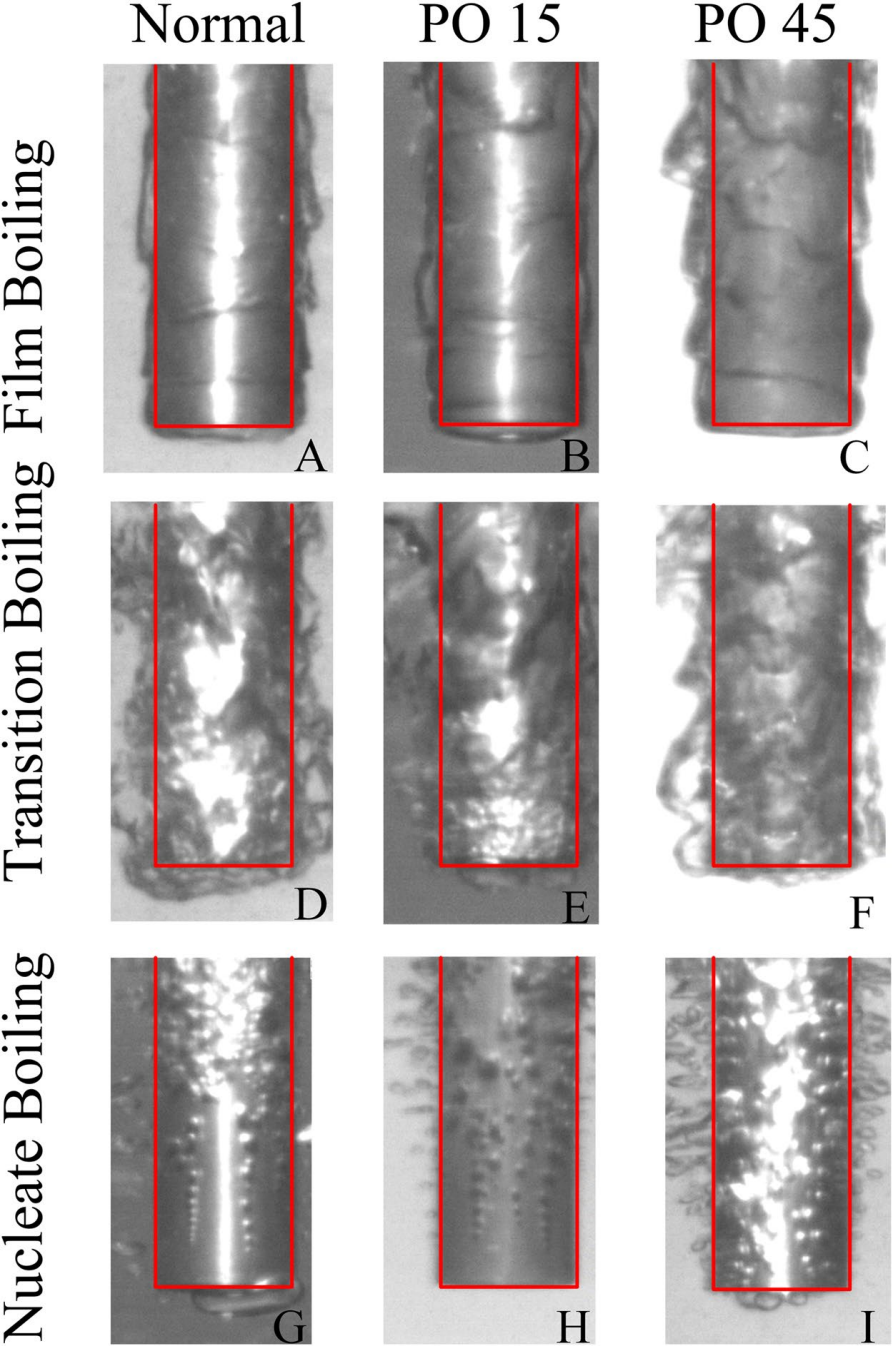
Comparison of the liquid-vapor flow patterns formed on conventional (left column) and nanoporous surfaces (middle and right columns) during liquid nitrogen quenching. (**A**,**B**,**C**) Film boiling regime; (**D**,**E**,**F**) Transition boiling regime; (**G**,**H**,**I**) Nucleate boiling regime.

Figure 7 provides images of the solid-fluid interactions on the normal and both nanoporous rod surfaces (PO15 and PO45) during the chilldown process in the three specific boiling regimes. The high-speed camera visualization shows how the liquid-vapor hydrodynamics and the resulting flow patterns along the surfaces evolve during chilldown. We found that distinct differences were observed among the three surfaces.

With respect to the film boiling regime in Fig. 7(A,B and C), the vapor film patterns on all three surfaces are very similar with one another. As discussed above, in the film boiling regime the surface is totally covered by thin vapor films such that the surface textures do not play any special role in the heat transfer. As a matter of fact, all three surfaces display the film boiling smooth wave patterns with various wavelengths.

In the transition boiling regime as shown in Fig. 7(D,E and F), the normal surface produced thicker and more chaotic two-phase patterns and the PO45 displays a much smoother and more organized two-phase flow pattern. However, the liquid-vapor two-phase layers on PO45 are relatively thicker than those in the film boiling, which is due to the fact that more vapor is generated as the liquid starts to contact the surface, resulting in higher rates of nucleation and bubble generation. The two-phase flow pattern on the PO15 surface is in between those of normal and PO45 surfaces. The reason that the PO45 has smoother liquid-vapor interfaces can be explained by the ‘micro-damper’ theory. The micro-damper novel concept was first introduced by Eroshenko and Fadeev^29, 30^. This concept, also called “nano colloidal damper”, is based on using colloidal suspension of microscale silica particles with nanoscale porous cavities as micro-dampers to replace the traditional hydraulic dampers. Eroshenko and Fadeev^29, 30^ explained that the effectiveness of damping is through the dissipative force of friction when water (working fluid) flowing through those nanoporous silica particles. Suciu *et al*.^31^ and Ku *et al*.^32^ further developed capillary transport theory for the colloidal damper and also carried out experiments and numerical modeling to verify the micro-damper concept introduced by Eroshenko and Fadeev^29, 30^. As in the current study, the experimental system used similar nanoporous surfaces, we therefore followed their verified concept and theory in explaining the nanoporous texture surface damping effects.

We assume that each nanoporous cavity is a hollow cylinder with radius r and length (depth) h. So the inner surface area of a hollow cylinder is 2*πrh*. Based on the PO15 and PO45 nano texture surface characteristic information given in Table 1, along with that Fig. 12(D) and (E) show that the depths of the nanoporous cavities for both surfaces are around 2 μm, the total effective surface area per cm^2^ flat surface area for PO15 is therefore estimated as,

$$2\times {10}^{10}cavities\times 2\pi \times 0.008\times {10}^{-4}(cm)\times 2\times {10}^{-4}(cm)=20.1\,c{m}^{2}$$

For PO45, it is estimated as,

$$1.24\times {10}^{10}cavities\times 2\pi \times 0.019\times {10}^{-4}(cm)\times 2\times {10}^{-4}(cm)=29.6\,c{m}^{2}$$

Since the normal surface has a surface area of 1 cm^2^, so the PO15 and PO 45 have approximately 20 and 30 times, respectively, more effective surface areas than that of the normal surface.

Millions of nanoscale porous cavities on the PO45 surface work as millions of micro-dampers that facilitate the attenuation of the high-frequency perturbations due to nucleation activities. As a result, perturbations cannot be amplified on the nanoporous surface, while the surface roughness would magnify and amplify the perturbations on the normal surface to form turbulent flows and interactions.

In the nucleate boiling regime, the two-phase flow pattern on the PO45 nanoporous surface shown in Fig. 7(I) indicates that a high density of larger and separated bubbles are generated in an explosive manner. However, as shown in Fig. 7(G) the normal surface results in a much lower bubble density and activity. The nanoporous structure on the PO45 surface results in longer attachment times for the bubbles on the surface, which is the main reason why the normal surface has lower heat fluxes in the nucleate boiling regime. The proper sized and patterned nanopores on the nanoporous surface alter the wettability, allowing more nucleation sites to be created that enhance nucleation and bubble escape from the surface.

### Analysis of boiling enhancement and modification by nanopores

**Figure 8:**
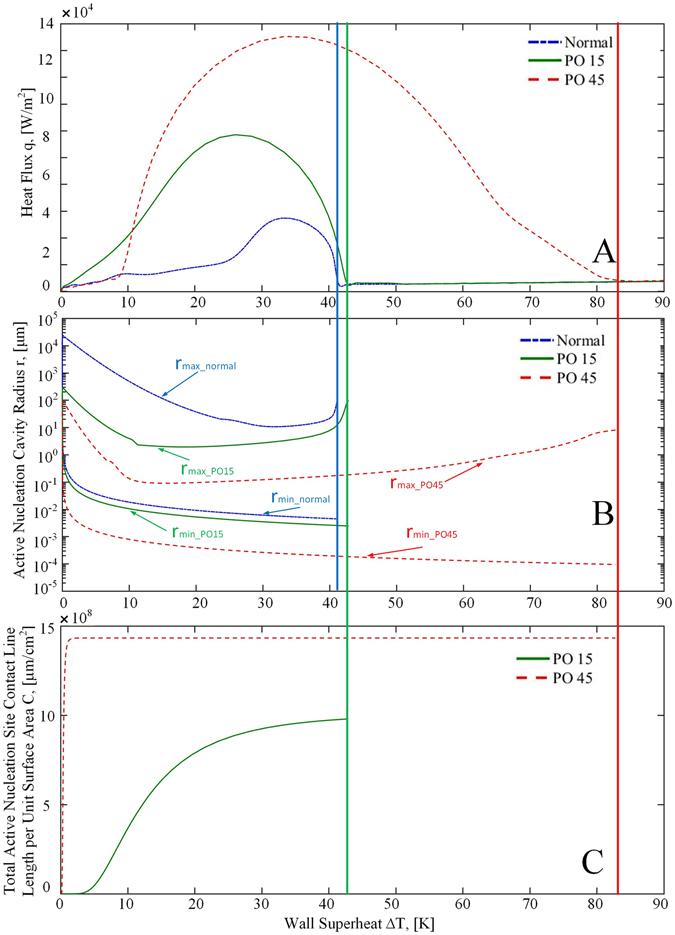
Active Nucleation cavity radii and total active nucleation site contact line lengths based on quenching boiling curve. (**A**) Boiling curve in transition and nucleate boiling regime. (**B**) Active nucleation site radius range (**C**) total circumference length of active nucleation sites per unit surface area.

**Figure 9:**
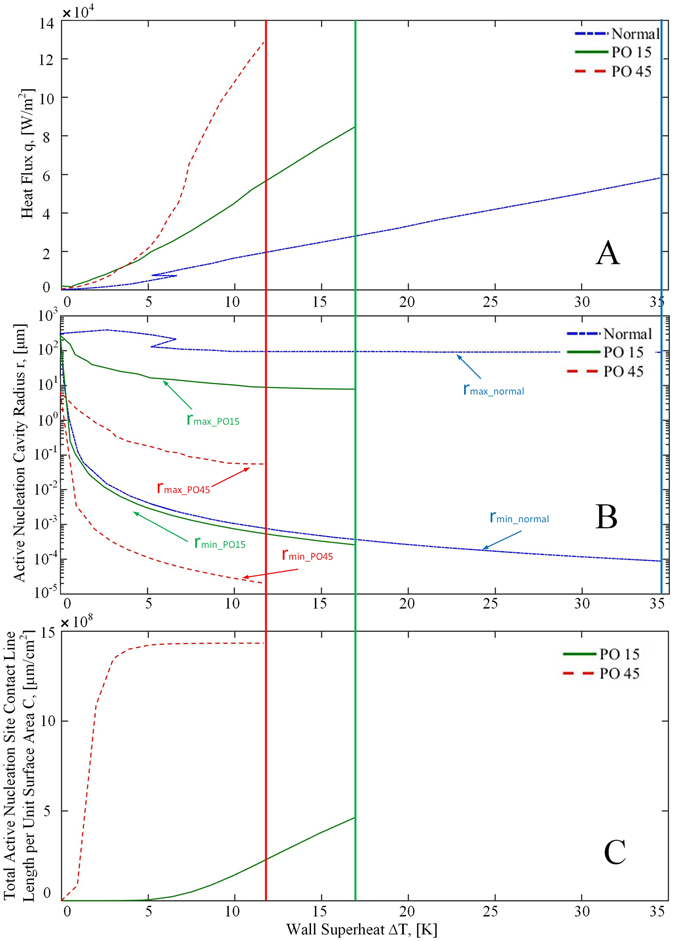
Active nucleation cavity radii range and total active nucleation site contact line lengths based on the heated boiling curve. (**A**) Boiling curve in the nucleate boiling regime. (**B**) Active nucleation site radius range (**C**) total circumference length of active nucleation sites per unit surface area.

In this section, we provide some quantitative analysis that relates the nanoporous surface textures to the heat transfer enhancements. The results are plotted in Figs 8 and 9 for quenching and heated boiling, respectively. The methods used for the detailed calculations of these surface nucleation parameters can be found in the Method section.

Figures 8(A) and 9(A) are the zoom-in plots of Figs 5 and 6 for transition and nucleate boiling regimes. The active nucleation site radius ranges as a function of the degree of wall superheat are plotted in Figs 8(B) and 9(B) for quenching and heated boiling conditions, respectively. It is noted that nucleation and bubble formation are only found in transition and nucleate boiling regimes during quenching while only in nucleate boiling for heated boiling. For quenching in the transition and nuclear boiling regimes, the minimum active nucleation site radius range, *r*_min_ varies from 5 × 10^−3^ µm to 1 µm for PO15 and from 10^−4^ µm to 10^−1^ µm for PO45. While the *r*_max_ varies from 2 µm to 5 × 10^2^ µm for PO15 and from 10^−1^ µm to 10^2^ µm for PO45. For heated boiling only in the nuclear boiling regime, the minimum active nucleation site radius range, *r*_min_ varies from 3 × 10^−4^ µm to 10^2^ µm for PO15 and from 2 × 10^−5^ µm to 5 µm for PO45. While the *r*_max_ varies from 1 µm to 3 × 10^2^ µm for PO15 and from 7 × 10^−2^ µm to 5 µm for PO45.

Before we can analyze the enhancement and modification on heated boiling and quenching heat transfer by nanoporous cavities, it is important to note that the wall surface heat fluxes in heterogeneous nucleate boiling are mainly due to the evaporation of liquid film that takes place in the nanoscale vicinity close to the moving contact lines of the bubbles where the vapor, liquid and solid meet one another according to a recent finding by Maroo and Chung^33^. As a result, the nucleate boiling heat flux is proportional to the total contact line length per unit surface area of all the active bubbles that are attached to the surface. According to Li *et al*.^28^, for liquids with large contact angles such as water, vapor bubbles tend to stay attached and grow out of the cavity mouth, and then depart from the smooth surface in the vicinity of the cavity mouth, while for cryogenic liquids with small contact angles, bubbles tend to detach and depart directly from the cavity mouth. As a result, the bubble departure diameter is larger than that of the cavity mouth for a large contact angle fluid, however for a cryogenic fluid, the bubble departure diameter is approximately equal to that of the cavity mouth. Therefore, the contact line length for the current liquid nitrogen fluid can be closely estimated by the perimeter of the nanopore of the active nucleation site. Based on the contact-line theory, using the nucleation site probability density function, the data of number density of nanopores on the surface (Fig. 2 and Table 1), and the ranges of active nucleation site radii (Figs 8(B) and 9(B)), the total active nucleation site contact-line length per unit surface area has been plotted for both the quenching and heating cases in Figs 8(C) and 9(C), respectively. As can be seen, the total contact line lengths for PO45 are generally more than twice of those for PO15 that would be verified and further discussed in the “Method” Section. The larger total contact-line length for PO45 supports its higher heat fluxes than PO15 as the magnitude of the transition and nucleate boiling heat fluxes are directly proportional to the total contact line lengths.

## Discussion

**Figure 10:**
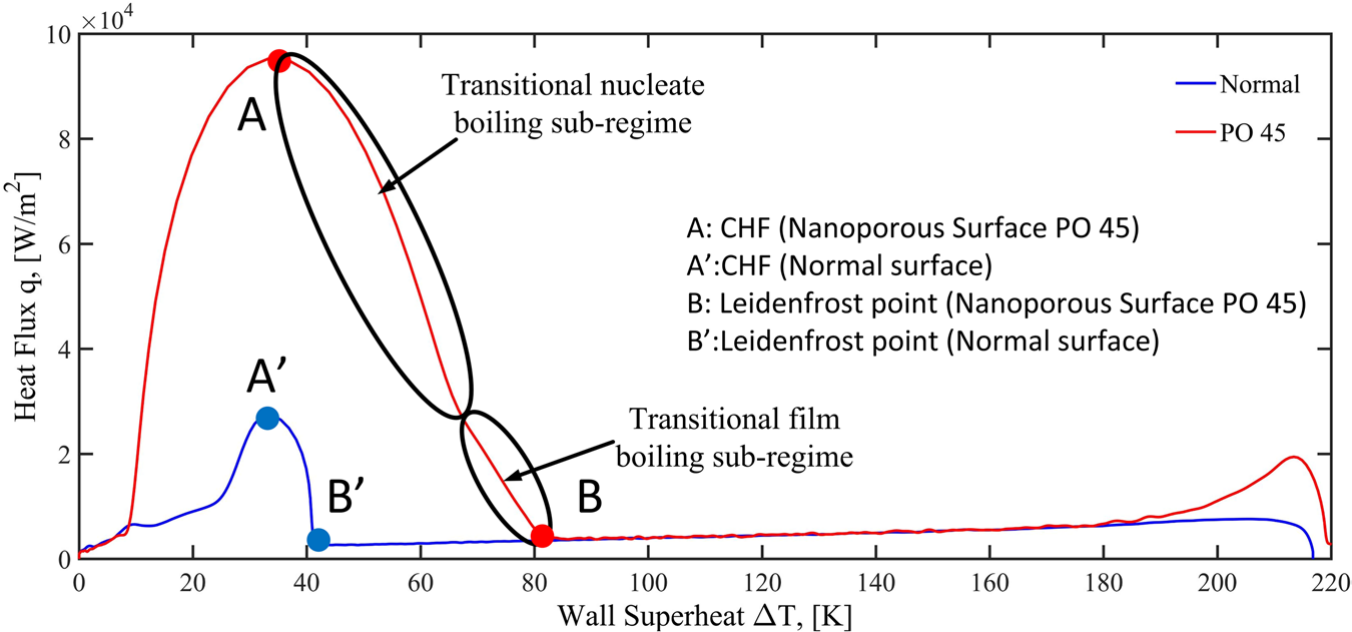
Comparison of the heat fluxes for nanoporous and conventional surfaces during quenching.

The two quenching curves (reversed boiling curves) obtained from the normal and PO45 nanoporous surface are plotted together in Fig. 10 to enlighten the effects produced by the nanoporous surface structures under identical experimental conditions (quenching fluid and its thermodynamic conditions, test rod geometry, orientation and position in the pool, and test rod material). Therefore, the differences between the two quenching curves are solely due to the nanoporous structures on the test rod surface. The large differences between the two quenching curves clearly demonstrate the ability of the nanoporous surface to modify the boiling process. We believe that this is the first time that the existence of different transition curves has been demonstrated experimentally even though Witte and Lienhard^34^ have conjectured this possibility. Their main concept is that the surface properties such as the surface nanoscale cavity characteristics and wettability can modify the nucleation and vaporization mechanisms that could enhance the heat transfer rates and accordingly the quenching curve characteristics. Next, the effects from the nanoporous surface are analyzed for each boiling regime.

As shown in Fig. 10, the PO45 nanoporous surface produces almost identical heat fluxes as those of the normal surface in the film boiling regime when the surface degree of superheat is higher than 82 K. At this point, the minimum heat flux (Leidenfrost point) is reached by the PO45 nanoporous surface. However, the normal surface did not reach the Leidenfrost point until the degree of surface superheat is 42 K. As a result, the PO45 nanoporous surface has produced a relatively large increase in the Leidenfrost temperature from 120 K to 160 K, a 40 K difference, corresponding to a 33% increase that shortens the film boiling regime substantially. Since the only difference between the two cases is the surface modification, we would attribute this change in the Leidenfrost temperature solely due to the increase in the nucleation and wettability by the superhydrophilic nanoporous surface as pointed out by Kruse *et al*.^14^.

According to Witte and Lienhard^30^ the Leidenfrost point on the normal surface is lower because the surface does not have enough nucleation capability and wettability for nucleation, as a result it has to wait until the surface is not hot enough to generate sufficient vapor to keep the film from collapsing that results in the rewetting of the surface. This type of surface rewetting that is also called the Leidenfrost phenomenon is based on the hydrodynamic instability suggested by Gottfried *et al*.^35^. However, the liquid is able to contact the surface on the nanoporous surface due to the enhanced wettability at a higher temperature, thus terminating the film boiling regime.

Comparing to the Leidenfrost point of the normal surface, Witte and Lienhard^34^ called the higher Leidenfrost temperature of the nanoporous surface a “jump” from the Leidenfrost temperature on the traditional normal surface. The Leidenfrost point on the normal surface is due to the phenomenon explained by the hydrodynamic theory that the surface is not hot enough to generate enough vapor to keep the film from collapsing. However, the reason for the “jump” on the nanoporous surface is owing to the enhanced wettability that enables the liquid to contact the surface at a higher temperature resulting in the ending of the film boiling.

As discussed in the above, the difference in heat transfer between the two surfaces is small in the film boiling regime (above 157 K) that is mainly due to the fact that the nanoporous surface basically makes the surface more hydrophilic that enhances the wettability and the nanoporous surface also provides more nucleation sites, both of those factors increase the rate of nucleation process. However, in film boiling, nucleation is not a contributing heat transfer mechanism.

The most significant modification to the boiling mechanisms by the nanoporous surface was found in the transition regime. If we examine the quenching curve of the nanoporous surface given in Fig. 10 for the transition regime, it is obvious that the curve is composed of two sub-regimes with different slopes connected at a surface temperature of about 145 K. Once again, Witte and Lienhard^30^ has provided some insights into this phenomenon and suggested that the lower heat flux sub-regime be called the transitional film boiling while the higher heat flux one be called the transitional nucleate boiling. The transitional film boiling sub-regime is essentially an extension of the film boiling regime where the film boiling mechanism is still dominant but limited liquid-solid contact can occur. Even the slightest liquid contact can lead to substantial nucleation and vapor generation due to the relatively higher surface temperatures. As the temperature of the surface decreases, more surface area can support the liquid contact and vapor generation that leads to a rise in heat flux. As the wall continues to cool down, at some point the surface area that allows liquid contact eventually overwhelms the dry area, moving the heat transfer process into the transitional nucleate boiling sub-regime. As the wall temperature continues to drop, more nucleation and vapor generation yield further increases in heat flux until the vapor escape path is no longer able to vent all the vapor generated, which corresponds to the CHF.

Compared to the normal surface, the nanoporous surface produced much higher heat fluxes in the transition boiling regime that is mainly due to the enhanced wettability and more nucleation cavity sites offered by the nanoporous surface. The enhanced wettability raised the Leidenfrost temperature that gives rise to relatively higher temperatures (more thermal energy potential) in the transition regime. The higher temperatures combined with more nucleation capability promote higher heat fluxes in the transition boiling regime.

As discussed above, CHF is simply the end point of the transition nucleate boiling sub-regime. The enhancement mechanism facilitated by the nanoporous surface described above results in a nearly 112% increase in CHF from 59 ± 3.1 to 125 ± 7.8 kW/m^2^. In the nucleate boiling regime, the surface is completely wetted and heat transfer is solely by nucleation and bubble generation. As mentioned above, the nanoporous surface is superhydrophilic and possesses extra nanoscale nucleation cavities that strongly enhance the nucleation and bubble generation rates, yielding much higher heat fluxes than those of the normal surface.

Based on the finding above, we conclude that the heat transfer modifications and enhancement are mainly attributed to the superhydrophilic surface property and billions of excessive nanoscale nucleation sites created by the nanoporous surface. Since these two characters are enabled by the nanoporous surface textures, and not caused by the boiling fluids, therefore we would suggest that similar results would be obtained if other media, such water, were employed as working fluids.

## Method

### System Design

**Figure 11:**
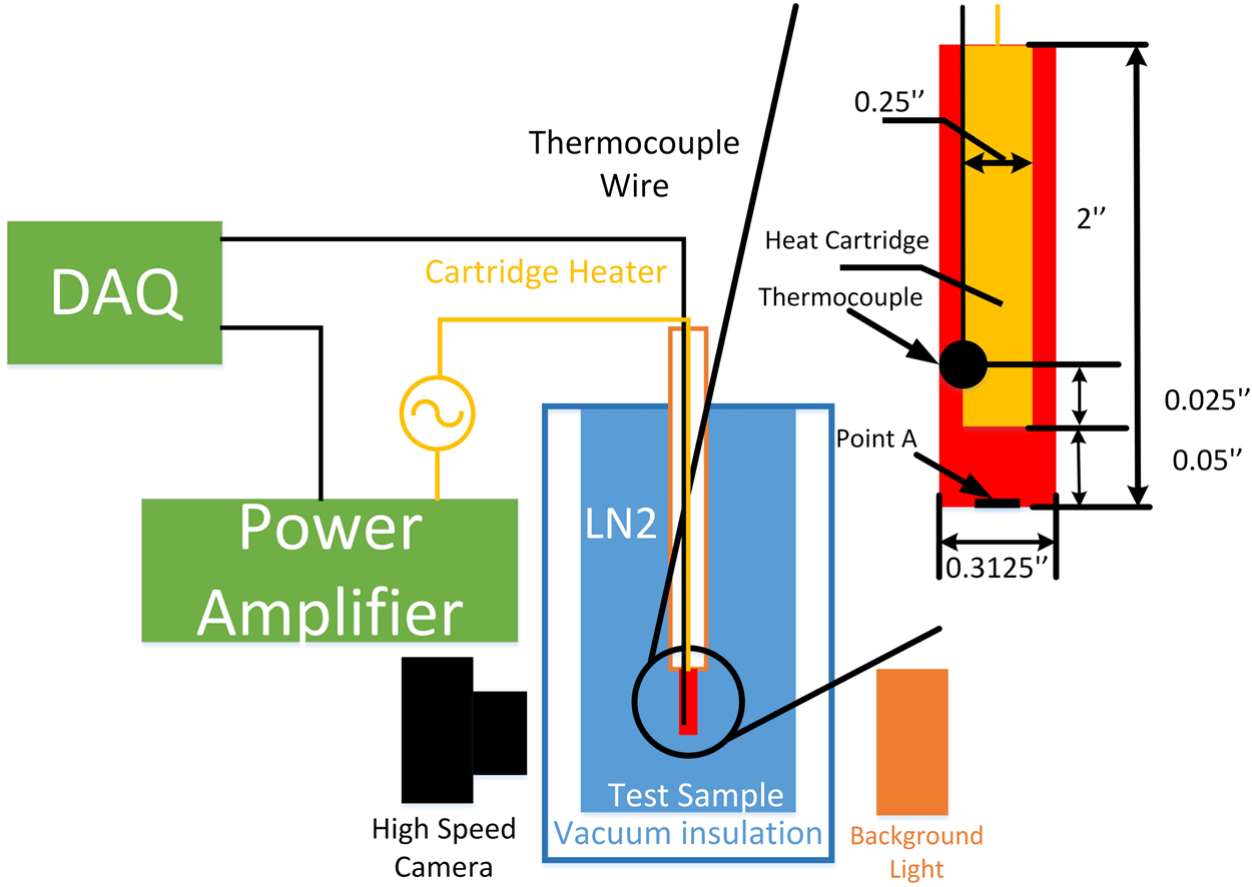
Schematic of experimental system.

An experimental apparatus was built with the purpose of exploring all the fundamental physics and transport mechanisms associated with boiling and quenching heat transfer while all the unessential boiling features were not included. Instead of an internal convective heat transfer system found in most engineering applications, a pool experiment was chosen since the basic characteristics of boiling are not fundamentally affected by the bulk flows. The basic schematic of the design is given in Fig. 11. A transparent glass Dewar with a vacuum jacket insulation for the walls was used to form a liquid nitrogen pool that was maintained at the atmospheric pressure through an opening to the atmosphere. The test section is a hollow cylindrical rod made of a 6061 multipurpose aluminum alloy which is nonmagnetic, resists stress cracking and has excellent resistance to corrosion. A fast response and high sensitivity T-type 40AWG (0.0799 mm diameter) micro thermocouple embedded in the bead was used to record the temperature history at 14 Hz data acquisition frequency. A heating cartridge is inserted into the center of the hollow test rod to provide heating during the boiling experiment. The electrical current signal applied to the heating cartridge is generated by the DAQ and amplified to the required power through a power amplifier. With a fixed gain factor, the DAQ generates different p-p values of 60 Hz sine wave currents. A set of background lights and a high-speed camera were introduced to collect the video data for the two-phase flow visualization. The video was used to analyze the patterns of the interaction between vapor and liquid phases. The recording speed was set at 2000 frames per second. To study the surface effect of nanoporous surfaces on boiling and quenching, the experiments were conducted on three different surfaces. One is a commercially available machine polished conventional aluminum surface, which is referred as the traditional surface, and the other two are aluminum surfaces coated with a different thin anodized aluminum oxide (AAO) nanoporous finish. The experiment was started when the room temperature (~293 K) test rod was dropped into the liquid nitrogen pool in the Dewar. When the rod is completely quenched to the temperature of 77.35 K, the quenching experiment is considered completed. Immediately after that, the heating cartridge was turned on to start the boiling experiment. Only steady state temperature and heating power data were recorded. At each heating level, the electrical current was maintained for 10 s in order to maintain a steady heating rate during the boiling experiment. In order to demonstrate the repeatability and reduce aleatory uncertainty, the same experimental procedure was repeated ten times on each AAO nanoporous test surface.

In order to maintain the property of surface to be constant for each experiment set, the surface has been given a detailed care. During the interval of experiments, the rod is heated by exposing it to the outside atmosphere and heated by air for 25 mins and cleaned by industrial nitrogen gas for 2 mins before each test to ensure the constant property on the surface. The repeatability has been maintained by 10 sets of tests for each surface structure.

### Uncertainty Analysis

The dimensions of the test rod were determined by a caliper. The mass of the test rod was measured through an electronic scale. The thermocouples had the uncertainty of ±1.5% full scale according to the manufacturer. Another uncertainty source for temperature measurements comes from the DAQ whose uncertainty was ±0.1% full scale. In the current experiment, the inverse heat conduction methods^1, 26, 27, 36, 37^ were used to obtain the surface heat fluxes from the measured temperatures. The uncertainty of the heat flux is calculated through a square root summation of partial derivatives. Both the uncertainties from the experimental apparatus and calculated parameters are listed in Table 3.

**Table 3:** Summary of the uncertainty.

Symbol	Uncertainty
d_i_ (mm)	±0.001 mm
M_r_ (g)	±0.0001 g
T_o_ (K)	±1.6%
T_i_ (K)	±1.6%
q_i_ (K)	±6.8%

The verification of the test facility is achieved by a close resemblance of the boiling curve of the normal surface in Fig. 10 with the standard boiling curve shown in Fig. 1.

### Preparation of Anodized Aluminum Oxide (AAO) Nanoporous Surfaces

**Figure 12:**
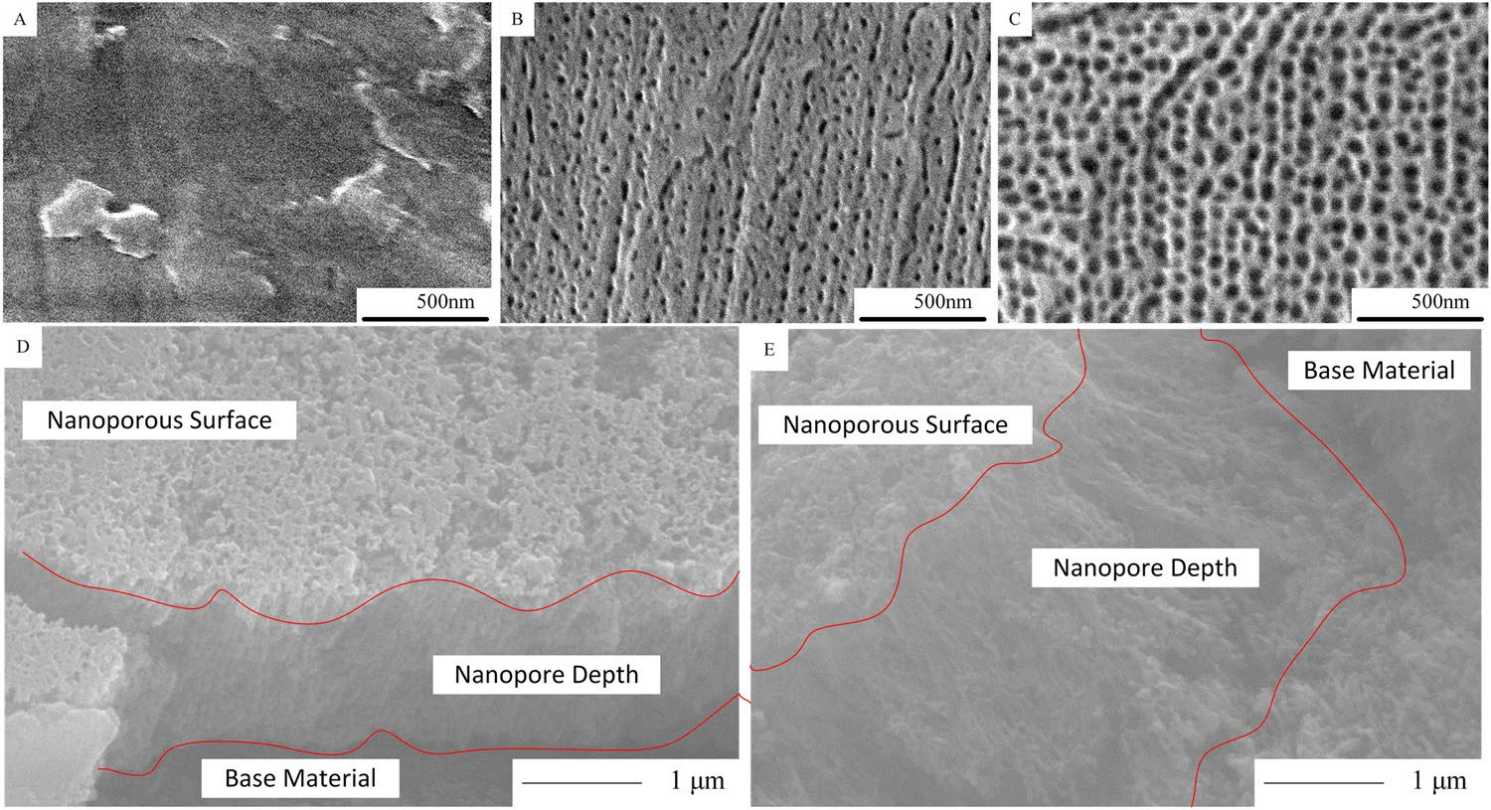
(**A**) Machine polished normal surface, (**B**) PO15 surface and (**C**) PO45 surface, (**D**) Cross-sectional view of PO15 and (**E**) Cross-sectional view for PO45.

The AAO nanoporous test surface was fabricated by a two-step anodization process. A pure aluminum rod was first cleaned by sonication in soapy de-ionized water, acetone, and ethanol, respectively. The cleaned aluminum rod was then put into an electropolish solution (Electro Polish System Inc.) at 65 °C and under a constant voltage of 17 V for 20 min. After that, the first anodization was carried out at 15 °C and under a constant voltage of 40 V in 0.3 M oxalic acid solution with stirring for 16 hours. After stripping off the AAO layer in a mixture of 10 wt% phosphoric acid and 1.8 wt% chromic acid, the second anodization was performed by the same procedure as that of the first. The processing time was varied to control the final AAO thickness to approximately 2 µm. At the end of the second anodization, the voltage was reduced at a rate of 0.5 V per 30 s to thin the barrier layer. The pore opening process was carried out in 5 wt% phosphoric acid at room temperature and was monitored by an electrochemical setup (VersaStat 3, Princeton Applied Research) using a small voltage of 0.1 V applied against a carbon counter electrode. In order to produce different nanopore sizes (diameters), the electrical current was applied for 15 minutes and 45 minutes, respectively for making two different nanopore size distributions, namely, PO15 and PO45, respectively. Figure 12(A) shows the image of machine polished normal surface. With an extra 30-minute electrochemical reaction time applied to the PO45 surface, the pore sizes are generally larger for PO45 than those of PO15 as shown in Fig. 12(B) and (C). Figure 12(D) and (E) show the in-depth cross-sectional views of PO15 and PO45 respectively. The depth for both of the two surfaces are around 2 μm. Both Fig. 12(D) and (E) are taken at the edges of the artificial scratches, where nanopores are eliminated and base material is exposed.

**Figure 13:**
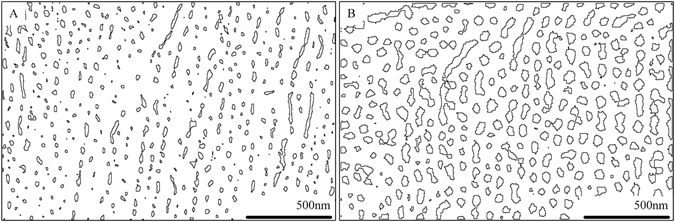
The digital image processed SEM photos for PO15 (left) and PO45 (right).

For the nanoporous surface structure made of 6061 aluminum alloy, the pore size distribution is no longer uniform but with a wider range of variations since there are different material elements involved besides aluminum. Therefore, knowing the detailed pore size distribution is essential for investigating the relationship between nanopores and phase change heat transfer. Using the established morphology boundary identification algorithm^38, 39^, the digital image processed SEM photos for test surfaces PO15 and PO45 are given in Fig. 13. The boundary lines of the nanopores are clearly shown.

In order to estimate the nanopore size distribution function for the test surface, a logarithmic function transformation is applied as below,

1 $$x={\mathrm{log}}_{10}(A)$$

where, A is the area occupied by the nanopores and x is the new introduced variable used for probability density function fitting. The area of the nanopores (A) can be calculated based on the binary figures shown in Fig. 13.

The Weibull distribution, Eq. (2) is used to simulate the distribution of nanopore sizes on the surface. K and λ are shape parameter and scale parameter for Weibull distribution, respectively. Similar method^35^ was used in porous material research.

2 $$f(r)=\{\begin{array}{c}\frac{k}{\lambda }{(\frac{x}{\lambda })}^{k-1}\exp (-{(\frac{x}{\lambda })}^{k})\\ 0\end{array}$$

The nanopore can be assumed to be circle. Therefore, Eq. (2) can be further simplified to be

3 $$f(r)=\{\begin{array}{c}\frac{k}{\lambda }{(\frac{{\mathrm{log}}_{10}(\pi {r}^{2})}{\lambda })}^{k-1}\exp (-{(\frac{{\mathrm{log}}_{10}(\pi {r}^{2})}{\lambda })}^{k})\\ 0\end{array}$$

Figure 2 shows the Eq. (3) and experimental data.

### Boiling Curve Reconstruction

For the study of liquid-to-vapor phase-change heat transfer, the so-called “boiling curve” (see Fig. 1) is often of great importance as it denotes the regimes of the different heat transfer mechanisms and their corresponding heat fluxes levels that are crucial design information for engineering applications. However, in order to construct the boiling curve, the transient temperatures and the corresponding heat fluxes of the test surface (Point A in Fig. 1) during the quenching and boiling experiment must be given. The surface temperature and the corresponding heat flux at point A were obtained by solving the inverse heat conduction problem (IHCP) using the temperature history recorded by the thermocouple (Point B in Fig. 1) embedded inside the test rod. The test section is approximated as a one-dimensional circular cylinder and the effect of thermal radiation is neglected. The details of the IHCP method is provided in our previous papers (Shaeffer *et al*.^1^, Yuan *et al*.^25^ and Hu *et al*.^27, 36, 37^).

### Modeling of Maximum and Minimum Active Nucleation Cavities

It is noted that the heat transfer rates are totally dependent on the rates of nucleation of bubble embryos in the nucleate boiling regime and while partially in the transition boiling regime. Therefore, it is legitimate to analyze the nucleation potentials for each test surface under transition and nucleate boiling conditions. Hsu^40^ proposed a model in the size range of active nucleation sites specified by the minimum radius (r_min_) and maximum radius (r_max_) of the active nucleate cavities (nucleation sites). The minimum radius (r_min_) and maximum radius (r_max_) are estimated based on the equation below.

4 $$\{{r}_{{\rm{\max }}},{r}_{{\rm{\min }}}\}=(\frac{{\delta }_{t}{C}_{2}}{2{C}_{1}})(\frac{{\rm{\Delta }}{T}_{w})}{{\rm{\Delta }}{T}_{w}+{\rm{\Delta }}{T}_{sub}})[1\pm \sqrt{1-\frac{8{C}_{1}\sigma {T}_{sat}({P}_{f})({\rm{\Delta }}{T}_{w}+{\rm{\Delta }}{T}_{sub})}{{\rho }_{v}{i}_{fg}{\delta }_{t}{({\rm{\Delta }}{T}_{w})}^{2}}}]$$

5 $${\rm{\Delta }}{T}_{sub}={T}_{sat}({P}_{f})-{T}_{f}$$

6 $${\rm{\Delta }}{T}_{w}={T}_{w}-{T}_{sat}({P}_{f})$$

7 $${C}_{1}=1+\,\cos \,\theta $$

8 $${C}_{2}=\,\sin \,\theta $$

9 $${\delta }_{t}=\frac{{k}_{l}}{h}$$

where h is the heat transfer coefficient on the wall, *k*_*l*_ is the thermal conductivity of liquid, *δ*_*t*_ is the thermal boundary layer thickness, θ represents the contact angle, C1 and C2 are coefficients defined as a function of surface contact angle. *T*_*sat*_(*P*_*f*_) is the saturated temperature with respect to the flow pressure *P*_*f*_. *T*_*f*_ is the flow temperature. σ is the surface tension with respect to flow pressure.
